# Cholecystoenterostomy or Choledochoenterostomy for Distal Bile Duct Obstruction?

**DOI:** 10.1155/1989/21271

**Published:** 1989

**Authors:** J. M. Little

**Affiliations:** Department of Surgery Westmead Hospital Westmead 2145 New South Wales Australia


9. Little, J.M. (1987) A prospective evaluation of computerized estimates of risk in the management of obstructive jaundice. *Surgery,* 473–6
10. Hawes, R.H., Cotton, P.B. and Vallon, A.G. Follow-up at six to eleven years after duodenoscopic sphincterotomy for stones in patients with prior cholecystectomy. *Gastroenterology* (in press)
11. Larson, R.E., Hodgin, J.R. and Priestly, J.T. (1966) The early and long-term results of 400 consecutive explorations of the common duct. *Surg Gyn Obst* **122**, 744–50

### CHOLECYSTOENTEROSTOMY OR CHOLEDOCHOENTEROSTOMY FOR DISTAL BILE DUCT OBSTRUCTION?

### ABSTRACT

*I. James Sarfeh, Eric, B. Rypins, James G. Jakowatz and George L. Juler.* (1988) A Prospective Randomised Clinical Investigation of Cholecystoenterostomy and choledochoenterostomy. The American Journal of Surgery, 155, 511–414.

**A prospective, randomized clinical trial was conducted to assess the efficacy of bilioenteric bypass in noncalculous distal biliary obstruction. Thirty-one patients required bypass for either malignant obstruction or chronic pancreatitis and were randomized into two groups: cholecystoenterostomy or choledochoenterostomy with cholecystectomy [15,16]. Nine bypasses failed after cholecystoenterostomy and two after choledochoenterostomy ($p < 0.04$). Eight of the 9 failures occurred in the subgroup of 22 patients with malignant biliary obstruction. In this subgroup, five bypasses failed within 90 days of operation, all after cholecystoenterostomy ($p = 0.03$ compared with choledochoenterostomy). The results indicate that choledochoenterostomy is the superior operation for malignant distal biliary obstruction. Additional studies will be necessary to identify the procedure of choice for benign noncalculous obstructions.**

### PAPER DISCUSSION

The authors are to be congratulated for conceiving and carrying out a prospective, randomized investigation of the values and defects of cholecystoenterostomy and choledochoenterostomy as alternative bypass conduits for patients with obstructive jaundice. All too often, opinions are given about the value of a method of treatment which are not based on good statistical evidence. Futhermore, surgeons are commonly criticized for not using prospective, randomized trials.

Thirty-one patients have been included in their trial, 15 undergoing cholecystenterostomy (CCE) and 16 choledochoenterostomy (CDE). Twenty-two of the patients had malignant jaundice, the remaining nine jaundice secondary to chronic pancreatitis. In the malignant group, 19 patients suffered from pancreatic carcinoma. A variety of other procedures, including gastroenterostomy, pancreaticoduodenectomy, colonic resection and gastrectomy were performed as necessary on selected patients. Patients were randomized according to whether the last digit of their social security number was odd or even. To be included in the trial, the patient needed to have evidence of a dilated common bile duct, a gallbladder and cystic duct distended with bile, no tumour encroachment on the end of the terminal cystic duct, no biliary tract stones and it was necessary that the surgeon believed that either CCE or CDE would be technically safe. The surgical technique was not standardised. All patients were followed post-operatively until death or the time of conclusion of the study. The bypass was considered to be a failure if further intervention was necessary for post-operative complications related to the biliary anastomosis or if obstructive jaundice recurred.

The authors planned to continue the study until a statistically significant difference had been demonstrated or until 50 patients had been randomized.

In summary, the authors found that CCE was quicker to perform and involved less operative blood loss. There was no difference in 30 day mortality between the groups. They found that post-operative complications were more frequent (8 in the CCE group, 3 in the CDE group, $P<0.05$). They also found that the failure rate was higher with CCE than with CDE (9 failures for CCE, 2 for CDE, $P<0.04$). The authors therefore conclude that CDE is a superior procedure, at least for malignant disease. There were too few patients with benign disease to reach any firm conclusion.

This study is a valuable one, but it has problems. These problems fall into three broad areas.

The first concerns the selection of patients. No attempt has been made to stratify the patients according to well known risk factors in obstructive jaundice.[1,2,3] Age and pre-operative total bilirubin are the only descriptive features apart from the diagnosis. There are well known stratification methods which would allow the reader to feel more confident that comparable groups had been included in each arm of the study.

The second selection factor which concerns me is the status of the cystic duct. The authors do not state how they assessed that the cystic duct was not compromised by tumour. That assessment is notoriously difficult at operation, since the point of insertion of the cystic duct is impossible to define by inspection and palpation. Radiological assessment will do better, and will give the surgeon some indication of the distance between the upper margin of the tumour and the lower margin of the cystic duct. There is a nagging doubt that patients in this study undergoing CCE may have been inappropriate for CCE on radiological assessment.

The second area of concern is the heterogeniety of the two groups. Although the diagnoses are similar in the two groups, and the additional procedures performed do not seem to differ, the methods of biliary anastomosis differ widely. Six patients in the CCE group had simple jejunal loops compared to none in the CDE group. In the CCE group, none had direct anastomosis to the duodenum whereas nine patient had choledochoduodenostomy. It is really quite difficult to justify comparing groups with such radical differences.

There seem to me also to be some statistical problems. I have recalculated the statistics, using Fisher's exact test as the authors did, with two tailed significance levels, and my results do not agree with theirs. For example, the bypass failure rate after CCE does not quite reach statistical significance, nor does the post-operative complication rate. I would point out also that there is an error in the summary, which states that "Nine bypasses failed after cholecystoenterostomy and two after choledochoenterostomy (P<0.04)." The text and Table 4 make it plain that 7 failures occurred after CCE. Statistically, therefore, the case against CCE becomes less clear.

There is one other point about the statistical analysis which deserves comment. In the text of the article, the authors state, "We predetermined that the study would conclude when differences in failure rates between the two operations reached statistical significance or when 50 patients were randomised." This implied that the authors repeatedly analysed their data to see whether statistical significance had been reached. It is well known that repeated analysis of data before a determined end point requires a change in significance level if a statistically significant difference to be accepted[4]. It the authors were concerned to reach their conclusion as quickly as possible, a properly designed sequential trial would have been the appropriate method to use[4] since they do not seem to have changed their significance levels to fit the repeated reanalysis.

I am left wondering whether the right question has been asked in this paper. It seems to me to be an appropriate decision to select the simplest applicable operation, particularly when dealing with patients who have a malignancy with a poor outlook. If the tumour is in fact well away from the entry of the cystic duct (say more than 1 cm.), then CCE seems to be a perfectly appropriate procedure. There are papers demonstrating that the failure rate of CCE under these circumstances is indeed very low.[5,6] If there is doubt about the relationship between the tumour and the entry of the cystic duct or if the tumour is definitely within 1 cm. of the entry of the cystic duct, then CDE is the appropriate procedure. CDE is definitely a more difficult operation and the authors have demonstrated this very well. It would be unfortunate if readers of this article went away with the idea that a clear cut case had been established for the complete abandonment of CCE in malignant disease. Our own experience of CCE in carefully *selected* patients has not been associated with the same morbidity nor the same rate of failure.[6] We would therefore continue to advocate a rational selection process rather than the adoption of CDE as a "routine" procedure.

**Keywords:** Cholecystoenterostomy, choledochoenterostomy, bile duct obstruction, cholestatic jaundice.

J.M. Little
Department of Surgery
Westmead Hospital
Westmead 2145, New South Wales
Australia

### REFERENCES

1. Pitt, H.A., Cameron, J.L., Postier, R.G. and Gadacz, T.R. (1981) Factors affecting mortality in biliary tract surgery. *American Journal of Surgery,* **141**, 66–72
2. Blamey, S.L., Fearon, K.C.H., Gilmour, W.H., Osborne, D.H. and Carter, D.C. (1983) Prediction of risk in biliary surgery. *British Journal of Surgery,* **70**, 535–38
3. Little, J.M. (1987) A prospective evaluation of computerized estimates of risk in the management of obstructive jaundice. *Surgery,* **102**, 473–76
4. Armitage, P. (1971) *Statistical methods in medical research.* Blackwell Scientific Publications, Oxford, Edinburgh, 415–425
5. Wongsuwanporn, T. and Basse, E. (1983) Palliative surgical treatment of sixty-eight patients with carcinoma of the head of pancreas. *Surgery, Gynecology and Obstetrics,* **156**, 73–75
6. Huang, J.F. and Little, J.M. (1987) Malignant jaundice. *Australian and New Zealand Journal of Surgery,* **57**, 905–909

